# Identification and quantification of lipids in wild and farmed Atlantic salmon (*Salmo salar*), and salmon feed by GC‐MS

**DOI:** 10.1002/fsn3.2911

**Published:** 2022-04-28

**Authors:** Eivind Molversmyr, Hanne Marie Devle, Carl Fredrik Naess‐Andresen, Dag Ekeberg

**Affiliations:** ^1^ 56625 Faculty of Chemistry, Biotechnology and Food Science Norwegian University of Life Sciences Ås Norway

**Keywords:** farmed, n‐3 fatty acids, nutrition, salmon, wild

## Abstract

The fatty acid profiles of wild and farmed Atlantic salmon (*Salmo salar*) and salmon feed was elucidated and quantitated. Due to the increasing proportion of vegetable oils in salmon feed, it was of interest to evaluate the effects on the farmed salmon fatty acid profile. There was found to be four times more fat in the muscle in farmed compared to wild salmon, 8.97 ± 0.63% and 2.14 ± 0.32%, respectively. The contents of saturated fatty acids, monounsaturated fatty acids, and polyunsaturated fatty acids were 15.0%, 55.4%, and 29.6%, respectively, in farmed salmon, while 26.3%, 47.4%, and 26.3% in wild salmon. The lipids were also fractioned into neutral lipids, free fatty acids, and polar lipids by solid‐phase extraction. Both wild and farmed salmon contained approximately equal amount of eicosapentaenoic acid and docosahexaenoic acid with 520 and 523 mg/100 g fish muscle, respectively. The salmons of both kinds were evaluated from a health perspective by discussing the contents of n‐3 and n‐6 fatty acids, saturated fatty acids, monounsaturated fatty acids, and polyunsaturated fatty acids together with nutritional quality indices. In conjunction with a lower fat intake by consumption, the wild Atlantic salmon displayed the most nutritionally beneficial profile.

## Graphical abstract

Both wild and farmed Atlantic salmon were evaluated from a health perspective by discussing the contents of n‐3 and n‐6 fatty acid, saturated fatty acids, monounsaturated fatty acids, and polyunsaturated fatty acids together with nutritional quality indices. Similar amounts of eicosapentaenoic acid and docosahexaenoic acid were found in both kinds, even though the wild counterpart had a significantly lower lipid content.

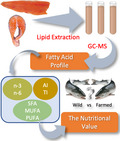

## 1 INTRODUCTION

The Atlantic salmon (*Salmo salar*) is a fish rich in lipids, in particular both eicosapentaenoic acid (EPA; C20:5n‐3c) and docosahexaenoic acid (DHA; C22:6n‐3c), and is one of the most important species in aquaculture in Europe, where Norway is the world's largest producer (Asche et al., 2019). However, there has been reported a decreased concentration of n‐3 fatty acids (FAs) in farmed salmon compared to the level in previous years (Aas et al., 2019). Due to the scarcity and increasing price of marine oils, the feed that previously consisted of 90% fish meal and fish oils have been reduced to 25%, while the rest has been substituted with plant‐based ingredients (Aas et al., 2019; Sprague et al., 2016). This substitution enabled a growth of 5.8% per annum in aquaculture production without a considerable increase in fish meal and fish oil consumption (Hamilton et al., 2020). In recent years in Norway, the proportion of plant‐based ingredients like plant oil and plant protein in the feed have increased. Recently, up to 2/3 of the lipid fraction in salmon feed is of rapeseed oil origin. In Norway today, the feed consists of 70% plant‐based ingredients as opposed to 60% in 2012 (Aas et al., 2019; Mørkøre et al., 2014). In contrast, the diet of wild salmon is based on small fish and crustaceans. Hence, the feed provided to farmed salmon differs from the natural diet (Renkawitz & Sheehan, 2011). This has profoundly altered the FA profile of farmed salmon and resulted in an approximate 50% reduction in the proportion of n‐3, and an increase in proportion of n‐6 FAs (FAO, 2018; Sissener, 2018; Sprague et al., 2016). The FA composition in salmon fillets have been shown to reflect that of the feed, possibly due to their limited ability to elongate and desaturate FAs (Sissener, 2018; Torstensen et al., 2006). This decrease in n‐3 FAs in fish feed can potentially have negative effects on both the fish health and that of the consumers (Rosenlund et al., 2016).

Throughout the years, many studies have been conducted to establish the importance of FAs on human health. By far the most extensively studied are the n‐3 long‐chained polyunsaturated fatty acids (PUFAs), which play a key role in human growth and development (Simopoulos, 1991). Both EPA and DHA are known to exhibit key roles in membrane functions, immunology, and inflammation, as well as prostaglandin metabolism (Simopoulos, 1991). Several diseases and disorders have been linked to deficiencies of DHA and n‐3 PUFAs. Namely, cardiovascular disease (CVD), attention deficit hyperactivity disorder, unipolar depression, and cystic fibrosis, among others (Horrocks & Yeo, 1999). Although both EPA and DHA can be produced by the human body, the rate of biosynthesis is low and insufficient, and they are recommended to be supplemented in the diet (Dewick, 2009). In 2012, the European Food Safety Authority (EFSA) set a recommendation of these marine n‐3 FAs in the range of 250–500 mg/day, or 1.75–3.50 g/week (EFSA Panel on Dietetic Products, 2012).

The dietary intake ratio of n‐6 to n‐3 FAs has also been reported to be of significance in overall health (Liu et al., 2017; Riediger et al., 2008; Russo, 2008; Yang et al., 2015). Apart from the n‐6/n‐3 ratio, two other nutritional quality indices, the atherogenicity (AI) and thrombogenicity index (TI), are commonly employed to estimate the nutritional value of PUFAs in human metabolism (Simopoulos, 2002; Ulbricht & Southgate, 1991). These indices are strongly associated with disease prevention and are claimed to promote health (Cherifi et al., 2018; Rhee et al., 2016; Simopoulos, 2002).

The main objective of this study was to determine and quantitate the FA levels in wild and farmed Atlantic salmon, with a focus on the saturated fatty acids (SFAs), monounsaturated fatty acids (MUFAs), PUFAs, n‐3 and n‐6 FAs, as well as the nutritional quality indices: AI, TI, and the n‐6/n‐3 ratio. This is evaluated in the context of nutritional differences by consumption of these two products. Additionally, the FA profile of salmon feed was also of interest to compare the similarities between the FA composition of farmed salmon and its feed.

## 2 MATERIALS AND METHODS

### 2.1 Chemicals and standards

The chloroform used for preparing the internal standards (ISs) and lipid extraction from the fish muscle samples was supplied by VWR Chemicals and was of Chromanorm quality (France). The methanol, used in conjunction with chloroform for the extraction procedure and to make the sodium methoxide solution, was supplied by Sigma‐Aldrich and was of Chromasolv quality (Poland). The derivatization of the lipids into fatty acid methyl esters (FAMEs) was performed using 14% BF_3_—methanol solution supplied by Sigma‐Aldrich (Switzerland). Heptane (≥99%) was supplied by Acros Organics (Belgium). The solution used to elute free fatty acids (FFA) by solid‐phase extraction (SPE) contained acetic acid and diethyl ether. The acetic acid 99.9% puriss p.a. was supplied by VWR Chemicals (France) and the diethyl ether puriss p.a. ≥99.8% was supplied by Sigma‐Aldrich (Poland).

A total of three different ISs: nonadecanoic acid (C19:0 FFA), trinonadecanoin (C19:0 TAG) and 1,2‐Dinonadecanoyl‐sn‐Glycero‐3‐phosphatidylcholine (C19:0 Pl), all supplied by Larodan AB (Malmö, Sweden), were chosen for quantitation of the FAMEs. The C19:0 TAG IS stock solution was prepared by dissolving 200 mg of standard with 20 ml of chloroform to a final concentration of 10 mg/ml. Both the C19:0 FFA and C19:0 Pl IS were prepared for two concentrations, 10 and 1 mg/ml. This was done by separately dissolving 20 mg of standard with 2 and 20 ml of chloroform, respectively. All IS stock solutions were transferred to GC vials, sealed, and stored in darkness at −20°C until use. A FAME‐mix containing 37 different components was used for the identification of FAMEs resulting from the derivatization of FA from the Atlantic salmon. The 37 Component FAME‐Mix was supplied by Supelco (Schnelldorf, Germany) and had a total concentration of 10 mg/ml. For further identification, 12‐methyl‐tetradecanoate, 13‐methyl‐tetradecanoate, *cis*‐7‐hexadecenoic acid methyl ester, *cis*‐11‐hexadecenoic acid methyl ester, all‐*cis*‐9,12‐hexadecadienoic acid methyl ester, *cis*‐6‐octadecenoic acid methyl ester, *cis*‐11‐octadecenoic acid methyl ester, *cis*‐13‐octadecenoic acid methyl ester, all‐*cis*‐6,9,12,15‐octadecatetraenoic acid methyl ester, *cis*‐9‐eicosenoic acid methyl ester, all‐*cis*‐8,11,14,17‐eicosatetraenoic acid methyl ester, all‐*cis*‐6,9,12,15,18‐heneicosapentaenoic acid methyl ester, and all‐*cis*‐7,10,13,16,19‐docosapentaenoic acid methyl ester were all purchased from Larodan AB (Malmö, Sweden).

### 2.2 Samples and sample preparation

The farmed Atlantic salmons (*n* = 3) were purchased fresh from a local fish market in Son, Norway, in August 2019. The sampled farmed salmons were all approximately the same size. Both the farmed salmon and the feed came from Vikenco AS located in Aukra (62°50′45″N, 6°46′34″E), Norway. The feed used in the production of the sampled farmed salmons was of the type “Rapid HF 1000 HQ 50A.” The feed sampled for this study was produced on November 17, 2019, by EWOS AS, Scotland. The wild salmons (*n* = 3) were acquired from Finnmarkfisk AS and were caught with salmon traps in Namsenfjorden (64°27′22″N, 11°30′09″E), outside of Namsen, Norway. The wild salmons came as packs of cutlets of 1 kg each and were frozen fresh at −20℃ since June 2019.

**FIGURE 1:**
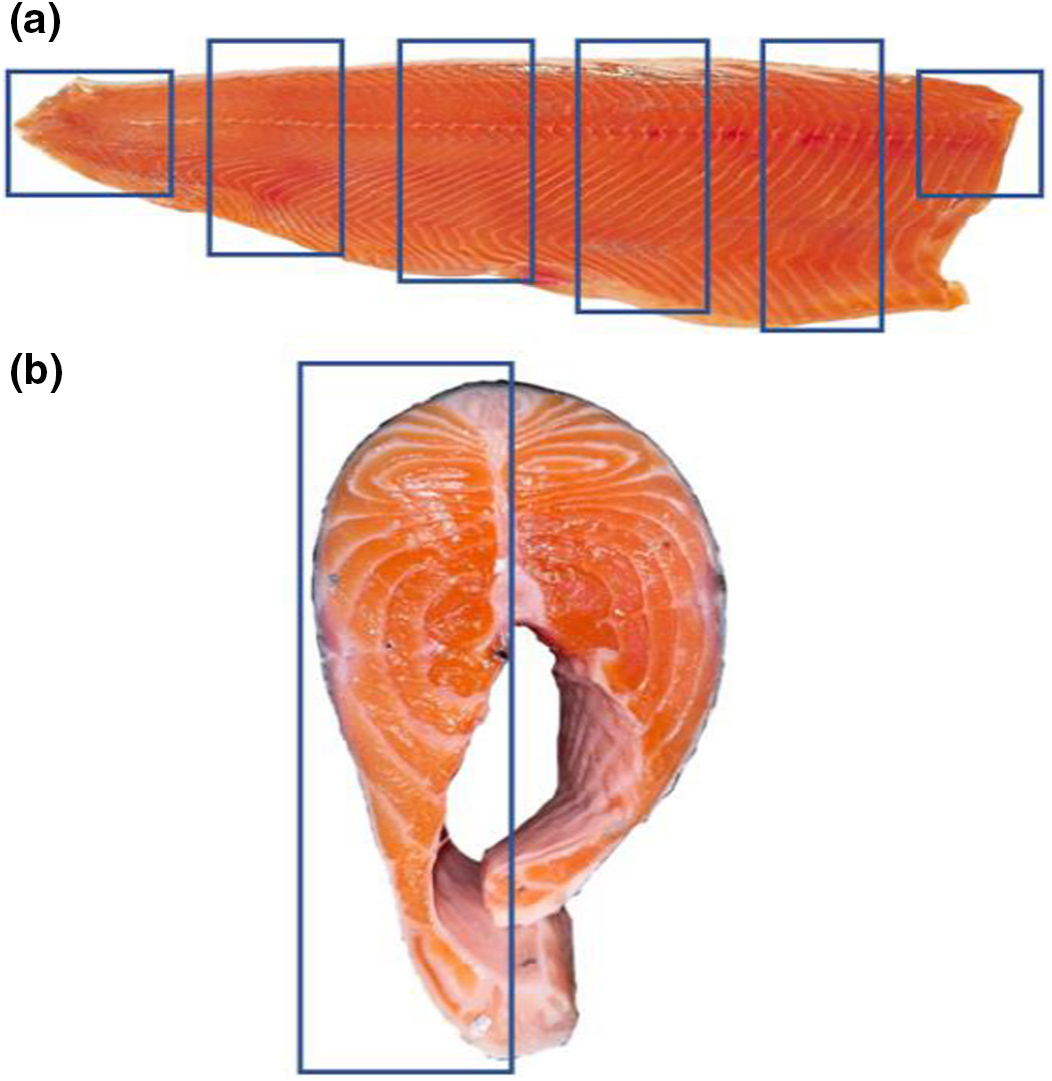
A diagram of: (a) salmon fillet in longitudinal section presenting the W‐shape of myomere and the two muscle types, and (b) the cross section of a salmon cutlet. The rectangles indicate the sampled areas

The farmed salmons were filleted, deboned, and deskinned. The subcutaneous fat was removed so only the fish muscle remained. Figure 1 shows a diagram of the muscles in both a salmon fillet (a) and cutlet (b). From the farmed salmon, both red and white muscles were sampled from all over the fillet as indicated by the blue rectangles in Figure 1a. The flesh was cut into smaller pieces and homogenized using a hand blender. This was done separately for each fish. The resulting muscle mass was stored in darkness at −20℃. The wild salmons came in the form of cutlets, but the same procedure for acquiring the muscle mass was used; however, one half of every cutlet in their respective packs were sampled as indicated in Figure 1b. The feed was delivered as pellets. The pellets were grinded into a homogenous mixture using a mortar. To keep the feed as fresh as possible, the pellets were grinded prior to the lipid extraction.

### 2.3 Lipid extraction procedure for determining lipid content

The lipids were extracted following Folch method (Folch et al., 1957). In brief, 3 g of homogenous muscle mass was transferred to 100 ml Erlenmeyer flasks, and added 60 ml of a 2:1 chloroform:methanol (v/v) solution. Lids were placed on top of the flasks, with subsequent shaking on an orbital shaker (Biosan PSU‐10i, Riga, Latvia) at 390 rpm for 30 min. The contents of the Erlenmeyer flasks were transferred to separatory funnels and added 12 ml of a 0.9% NaCl in Milli‐Q water solution. Chloroform was used to wash the flasks for any lipid residues. The separatory funnels were shaken vigorously until satisfactory separation of the two phases was achieved, and the lower organic phase was transferred to 120 ml Büchi reagent tubes. Two additional liquid–liquid extractions were carried out with 10 ml chloroform and collected in the same reagent tubes. The collected organic phase was dried using a vacuum evaporator system (Büchi, Syncore^®^ Polyvap equipped with a V‐700 vacuum pump and a V‐855 vacuum controller) at 40℃, 100 rpm, and an air pressure of 207 mbar. When most of the solvent was evaporated, the content was transferred to preweighed culture tubes (DURAN^®^, GL14). The complete removal of solvent was carried out by inserting the tubes in heating blocks at 40℃ under pure nitrogen flow. The dry residues were weighed to calculate the total lipid content of the fish.

### 2.4 Lipid extraction and derivatization into FAMEs

Different volumes of C19:0 TAG ISs were added in two series to allow quantitation of the compounds in the chromatogram. The added volumes for the 1st series were 200 µl and 100 µl for farmed and wild salmon, respectively, while in the 2nd series were 50 µl and 10 µl for farmed and wild salmon, respectively. The salmon feed shared the same added volumes as the farmed salmon.

To a 50 ml screw cap tube (Greiner Bio‐One, Cellstar^®^ Tubes), 0.5 g muscle mass was added in two series of four parallels each. IS and 10 ml of a 2:1 chloroform:methanol (v/v) solution was added and shaken at 390 rpm for 20 min using an orbital shaker. Then, 2 ml of a 0.9% NaCl in Milli‐Q water solution was added and shaken using a vortex mixer (IKA^®^‐Werke, Yellowstone TTS‐2). The two phases were then separated by centrifugation (Beckman CoulterTM, AvantiTM J‐25 equipped with a JA‐12 fixed‐angle rotor), 5 min at 716 rcf. The upper aqueous phases were discarded, and the organic phases were transferred to test tubes. The samples were heated to 40℃ under nitrogen flow until dryness. The complete removal of solvent was carried out by inserting the tubes in heating blocks at 40℃ under pure nitrogen flow. The dry residues were resolved in 1 ml of n‐heptane and transferred to culture tubes. A sodium methoxide solution was prepared by dissolving metallic sodium, supplied by Merck (Darmstadt, Germany), in methanol to a final concentration of 5 mg/ml. To each of the culture tubes, 1 ml of the sodium methoxide solution was added, followed by horizontal shaking using an orbital shaker at 390 rpm for 30 min. One milliliter of 14% BF_3_‐methanol solution was added to each of the culture tubes and heated in a water bath at 80℃ for 20 min. The tubes were cooled to room temperature and the two phases were separated by centrifugation (Hettich^®^, EBA 20) for 5 min at 2000 rpm (381 rcf). The heptane phase was transferred to GC vials and diluted with n‐heptane. The wild salmon samples were diluted 1:10, the farmed salmon samples were diluted 1:50, and the salmon feed samples were diluted 1:100. The samples were stored in darkness at −20 ℃ until analysis with GC‐MS.

### 2.5 Solid‐Phase extraction and methylation

The lipids were extracted in two series of four parallels each, following the same procedure as the previous section; however, three different ISs were added. The added volumes for the 1st series were 200 and 100 µl of C19:0 TAG, 15 and 10 µl of C19:0 FFA (10 mg/ml), and 50 and 25 µl of C19:0 Pl (10 mg/ml), for farmed and wild salmon, respectively. The added volumes for the 2nd series were 20 and 10 µl of C19:0 TAG, 15 and 10 µl of C19:0 FFA (1 mg/ml), and 50 and 25 µl of C19:0 Pl (1 mg/ml), for farmed and wild salmon, respectively. Furthermore, the dry extracted lipids were resolved in 1 ml of chloroform and transferred to GC vials. Blank samples of pure chloroform were also prepared. The samples were stored in darkness at −20℃ until fractioning.

The method using solid‐phase extraction (SPE) for lipid fractionation was based on the previous works of Pinkart et al. (1998) and Ruiz Carrascal et al. (2004) and was carried out using a GX‐274 ASPEC™ (Gilson, Middleton, WI, USA) and the accompanying software TRILUTION^®^ LH Software v.3.0 (Gilson, Middleton, WI, USA). Due to a storage shortage, two different columns were used as the stationary phase for the different series. Discovery DSC‐NH_2_ 500 mg and 3 ml columns (Sigma‐Aldrich, USA) were used in the 1st series, while Bond‐Elut NH_2_ 500 mg and 3 ml columns (Agilent Technologies, USA) were used in the 2nd series. The columns were conditioned using 7.5 ml heptane and a flow rate of 1.0 ml/min, prior to the application of the samples (500 µl). The neutral lipids (NLs) were eluted into glass vials using 5.0 ml chloroform, the FFA using 5.0 ml of a 98:2 diethyl ether:acetic acid (v/v) solution, and the polar lipids (PL) using 5.0 ml of methanol. The contents of the glass vials were transferred to culture tubes. Chloroform was used to wash the glass vials for any lipid residues. Blank samples were prepared and analyzed for both column types. In both columns, the FFA fraction showed a contribution of C14:0, C16:0, and C18:0. To compensate for this, the mean areas of the contributions were subtracted from their respective counterparts in the FFA samples.

The complete removal of solvent was carried out by inserting the tubes containing the three fractions and blanks into heating blocks at 40℃ under nitrogen flow to dryness. The methylation procedure followed the same procedure as in the previous section, with some modifications. The dry residues of the NL and PL fractions were resolved in 2 ml n‐heptane, added 1.5 ml of sodium methoxide (5.0 mg/ml), and horizontally shaken at 390 rpm for 30 min using an orbital shaker. To separate the two phases, the tubes were left in vertical position for 30 min. The dry residues of the FFA fraction were added 1 ml of 14% boron‐trifluoride‐methanol solution and heated for 5 min at 80℃ in a water bath. The tubes were cooled to room temperature, added 2 ml n‐heptane, and shaken by a vortex mixer. The tubes were left in vertical position for 15 min. The upper heptane phases of all fractions were transferred to GC vials and stored in darkness at −20℃ until analysis with GC‐MS. The NL fractions of farmed salmon were diluted 1:10 with n‐heptane.

### 2.6 Analysis of FAMEs by GC‐MS

An ISQ^TM^ QD GC‐MS (Thermo Fisher Scientific, Waltham, MA, USA) was used to identify the FAMEs in the samples. The MS was a single quadrupole instrument. Electron ionization was used as the ionization method (70 eV electrons), and a mass range of *m/z* 50–600 was chosen. Both the ion source and transfer line were kept at a temperature of 250°C. Full‐scan acquisition mode was utilized with a scan time of 0.2 s.

The GC used in combination with the MS was a TRACE^TM^ 1310 (Thermo Fisher Scientific, Waltham, MA, USA), equipped with a 60 m Rtx^®^‐2330 column with an inner diameter of 0.25 mm and a 0.2 µm film thickness of fused silica biscyanopropyl cyanopropylphenyl polysiloxane stationary phase (Restek, Bellefonte, PA, USA). To inject the sample, an AI/AS 1310 Series Autosampler was utilized (Thermo Fisher Scientific, Waltham, MA, USA), injecting 1.0 μl at a split ratio of 1:10 into an injection chamber set to 250°C, using helium as carrier gas (99.99990%, from Yara, Rjukan, Norway) at a constant flow of 1.0 ml/min. The total run time was set to 110 min, with the initial GC oven temperature set to 50°C for 5 min, before increasing, at a rate of 100°C/min, to 140°C and held for 30 min. The temperature was increased to 145°C, at a rate of 10°C/min and held for 30 min, before increased further, at a rate of 3°C/min, to 175°C and held for an additional 20 min. Finally, at a rate of 50°C/min, the temperature was held at 260°C for 10 min.

For the identification and quantitation of the complete FA profiles, a single injection of each diluted quadruplicates was subjected to analysis by GC‐MS. A single injection of n‐heptane was carried out in‐between samples replicates of different fish. For the samples prepared using off‐line SPE, undiluted quadruplicates were made for each of the following fractions: NL, FFA, and PL for both kinds of salmons, except for the NL fractions of the farmed salmon which was diluted. Undiluted quadruplicates were also prepared for the three fractions of the blank samples. A single injection was carried out for each sample replicate, with one injection of heptane in‐between samples replicates of different fish. The software used for the GC‐MS analysis was Chromeleon v7.2.8 (Thermo Fisher Scientific, Waltham, MA, USA). For the identification of FAME, NIST 17 Mass Spectral Library (Gaithersburg, MD, USA) was used in conjunction with the retention times of the independent standards as well as the standards present in Supelco 37 Component FAME‐Mix.

### 2.7 Nutritional quality indices of the lipids

To estimate the nutritional quality of the lipids, two separate indices were to be calculated in addition to the n‐6/n‐3 ratio. The AI and TI were calculated by using the empirical equations, Equations 1 and 2, respectively, according to Ulbricht and Southgate (Ulbricht & Southgate, 1991).

(1) $$\mathrm{AI}=\frac{\left[C12:0\;+\;\left(4*C14:0\right)\;+\;C16:0\right]}{\left(\sum MUFAs\;+\;\sum n‐6\;+\;\sum n‐3\right)}$$

(2) $$\mathrm{TI}=\frac{\left[C14:0\;+\;C16:0\;+\;C18:0\right]}{\left[\left(0.5*\sum MUFAs\right)+\left(0.5*\sum n‐6\;+\left(3*\sum n‐3\right)\;+\left(\frac{\sum n‐3}{\sum n‐6}\right)\right)\right]}$$

## 3 RESULTS AND DISCUSSION

### 3.1 Lipid content

The average total lipid content of the farmed salmon muscle was found to be four times that of the wild salmon (8.97 ± 0.63% and 2.14 ± 0.32%, respectively). There have been several studies done on this subject. Although a previous study done by Blanchet et al. (2005) found that the lipid content of the two types of salmon were approximately the same, more recent studies have found that the lipid content is much higher in farmed salmon (Jensen et al., 2012; Lundebye et al., 2017). This was also the case for our study. However, both Jensen et al. (2012) and Lundebye et al. (2017) reported average lipid content in the range of 12%–14% and 6%–8% for farmed and wild salmon, respectively, and thus report higher values than our results. Apart from the biological factors and individual differences, these are believed to originate from differences in sampling methods. Both Jensen et al. (2012) and Lundebye et al. (2017) sampled the salmon following the Norwegian Quality Cut, where only the flesh cut between the dorsal and adipose fin and down to the gut is sampled. Furthermore, the subcutaneous fat is not removed. Our study focused on determining the lipid content in fish muscle and we deemed it appropriate to remove the subcutaneous fat and sample cuts from the entire fish to get a representative muscle sample. The wild salmon had been frozen since June 2019 and, albeit being frozen fresh and stored in the freezer, some of the FAs may have been oxidized (Dawson et al., 2018). The lipid extractions were performed in early August 2019. Several researchers have reported that the lipid content decrease due to oxidation during storage in the freezer; however, due to the short storage period, this effect of lipid oxidation is limited (Arannilewa et al., 2005; Gandotra et al., 2012; Kamal et al., 1996; Omotosho & Olu, 1995; Refsgaard et al., 2000). Even so, the results could have been better comparable if both farmed salmon and wild salmon had been fresh. However, most commercially available fish products have been frozen at some point, so these results might offer the most relevant picture for the nutritional values. Based on the present study, and assuming that a dinner portion of fish fillet is 200 g, one would receive 4.3 g of fat from wild salmon and 17.9 g of fat from farmed salmon. Thus, consuming farmed salmon results in a higher fat intake.

### 3.2 FA profile of wild and farmed Atlantic salmon and salmon feed

The FA composition of the muscles of wild and farmed salmon together with the composite values for the feed given to the farmed salmon are provided in Table 1. By utilizing Equations 1 and 2, the AI and TI values were calculated and are included in Table 1. A total of 36, 35, and 34 FAs were found in wild salmon, farmed salmon, and salmon feed, respectively. This implies that we found 39 unique FAs, where C12:0 being the shortest and C24:1n‐9c the longest FA. All the unsaturated FAs found exhibited a cis configuration. The FA composition is mainly reflected by the FA composition of the feed (Jensen et al., 2012). As the feeding regime is widely different for the farmed and wild salmon, it was expected to be reflected in the FA profiles. Compared to the wild salmon, four FAs in particular stand out in the FA profile of the farmed salmon. C16:0, oleic acid (OA; C18:1n‐9c), linoleic acid (LA; C18:2n‐6c), and alpha‐linolenic acid (ALA; C18:3n‐3c) are present in relatively high concentrations (482–3,756 mg/100 g fish muscle) and together accounted for 73% of the FAs in farmed salmon. In the wild counterpart, these four FAs exist at lower concentrations and constitute only 36% of the total FA content. ALA is the precursor to both EPA and DHA, and, along with LA, make up the essential fatty acids (EFAs) which are needed to be incorporated in the diet (Dewick, 2009). In total, these two EFAs make up 19.5% of the FA content in farmed salmon, whereas only 1.5% in wild salmon. OA, LA, and ALA are found in greater concentrations in farmed salmon compared to the wild counterpart.

**TABLE 1:** FA composition (% of total FA content) and amount of FA (mg per 100 g of muscle) in farmed (*n* = 3) and wild (*n* = 3) Atlantic salmon and salmon feed (*n* = 4)

Fatty acid	Wild salmon		Farmed salmon		Feed	
	Composition (%)	Amount (mg/100 g)	Composition (%)	Amount (mg/100 g)	Composition (%)	Amount (mg/100 g)
C12:0	0.05 ± 0.01^a^	1.38 ± 0.15	n.d.	n.d.	n.d.	n.d.
C13:0 (4,8,12‐trimethyl)^b^	0.063 ± 0.004	1.71 ± 0.12	n.d.	n.d.	n.d.	n.d.
C14:0	3.43 ± 0.49	93.7 ± 13.2	1.71 ± 0.42	145.5 ± 35.0	2.18 ± 0.09	653.1 ± 26.2
C14:0 (13‐methyl)	0.14 ± 0.02	3.71 ± 0.53	0.03 ± 0.01	2.71 ± 0.62	0.07 ± 0.01	19.53 ± 1.69
C14:0 (12‐methyl)	0.09 ± 0.01	2.33 ± 0.35	0.018 ± 0.004	1.57 ± 0.33	0.027 ± 0.002	8.04 ± 0.50
C15:0	0.30 ± 0.05	8.13 ± 1.42	0.09 ± 0.02	7.94 ± 1.75	0.19 ± 0.01	56.11 ± 3.32
C16:0	17.43 ± 2.18	475.7 ± 59.5	9.61 ± 2.22	819 ± 189	10.32 ± 0.30	3,097.4 ± 91.5
C17:0	0.43 ± 0.05	11.67 ± 1.27	0.18 ± 0.04	15.44 ± 3.77	0.34 ± 0.03	101.22 ± 8.77
C18:0	4.31 ± 0.56	117.7 ± 15.4	2.94 ± 0.75	250.4 ± 64.1	4.37 ± 0.13	1,312 ± 38.9
C20:0	0.08 ± 0.01	2.22 ± 0.29	0.29 ± 0.08	24.54 ± 6.68	0.49 ± 0.02	146.34 ± 7.20
C22:0	n.d.	n.d.	0.09 ± 0.01	7.81 ± 0.69	0.23 ± 0.02	70.27 ± 4.88
C24:0	n.d.	n.d.	0.035 ± 0.005	2.95 ± 0.42	0.119 ± 0.004	35.71 ± 1.10
**∑ SFA**	**26.32 ± 3.38**	**718.2 ± 92.3**	**14.98 ± 3.55**	**1,278 ± 303**	**18.33 ± 0.61**	**5,500 ± 184**
C16:1n‐9c	0.13 ± 0.03	3.66 ± 0.83	0.11 ± 0.03	9.63 ± 2.73	0.09 ± 0.01	25.77 ± 2.45
C16:1n‐7c	6.39 ± 1.26	174.5 ± 34.5	2.57 ± 0.66	219.1 ± 56.1	3.12 ± 0.11	935.8 ± 32.2
C16:1n‐5c	0.19 ± 0.03	5.14 ± 0.73	n.d.	n.d.	n.d.	n.d.
C17:1n‐7c	0.26 ± 0.05	6.95 ± 1.25	0.08 ± 0.02	6.78 ± 2.02	0.09 ± 0.01	27.50 ± 2.23
C18:1n‐12c	0.78 ± 0.27	21.32 ± 7.26	0.12 ± 0.06	10.25 ± 4.71	n.d.	n.d.
C18:1n‐9c	17.14 ± 2.24	467.7 ± 61.0	44.0 ± 11.1	3,756 ± 943	41.42 ± 1.15	12,427 ± 345
C18:1n‐7c	3.86 ± 0.19	105.46 ± 5.12	3.00 ± 0.80	256.3 ± 68.6	2.96 ± 0.09	887.6 ± 26.8
C18:1n‐5c	0.22 ± 0.03	6.12 ± 0.75	n.d.	n.d.	n.d.	n.d.
C20:1n‐11c	0.79 ± 0.14	21.54 ± 3.89	0.14 ± 0.06	11.88 ± 4.81	0.13 ± 0.01	37.60 ± 2.67
C20:1n‐9c	8.05 ± 1.76	219 ± 48.1	3.43 ± 1.13	292.3 ± 93.6	1.95 ± 0.06	583.9 ± 17.3
C20:1n‐7c^b^	0.23 ± 0.09	6.31 ± 2.28	0.09 ± 0.02	7.47 ± 2.00	0.09 ± 0.01	26.48 ± 1.54
C22:1n‐9c	8.85 ± 2.24	241.5 ± 61.1	1.46 ± 0.73	124.8 ± 61.9	1.17 ± 0.05	352.5 ± 14.0
C24:1n‐9c	0.50 ± 0.04	13.50 ± 1.05	0.36 ± 0.13	30.3 ± 11.2	0.21 ± 0.01	61.57 ± 1.87
**∑ MUFA**	**47.40 ± 8.36**	**1,293 ± 228**	**55.4 ± 14.7**	**4,725 ± 1,254**	**51.22 ± 1.49**	**15,366 ± 446**
C16:2n‐4c	0.24 ± 0.08	6.62 ± 2.17	0.14 ± 0.04	11.58 ± 3.06	0.28 ± 0.02	83.11 ± 5.77
C18:2n‐6c (LA)	0.84 ± 0.14	22.94 ± 3.90	13.83 ± 3.33	1179 ± 284	13.86 ± 0.39	4157 ± 117
C18:3n‐6c	n.d.	n.d.	0.06 ± 0.02	5.54 ± 1.99	0.053 ± 0.004	16.04 ± 1.32
C18:3n‐3c (ALA)	0.64 ± 0.09	17.51 ± 2.34	5.66 ± 1.10	482.9 ± 94.2	7.99 ± 0.23	2,396.5 ± 70.5
C18:4n‐3c	0.86 ± 0.03	23.58 ± 0.93	0.43 ± 0.10	36.29 ± 8.55	0.56 ± 0.04	167.5 ± 11.7
C20:2n‐6c	0.21 ± 0.06	5.71 ± 1.63	0.84 ± 0.22	71.5 ± 19.1	0.09 ± 0.01	26.17 ± 2.38
C20:3n‐6c	0.06 ± 0.02	1.54 ± 0.43	0.16 ± 0.04	14.00 ± 3.59	0.044 ± 0.004	62.00 ± 1.06
C20:3n‐3c	0.15 ± 0.04	3.98 ± 1.15	0.34 ± 0.07	29.27 ± 6.20	0.036 ± 0.001	10.67 ± 0.29
C20:4n‐6c	0.26 ± 0.05	7.17 ± 1.34	0.13 ± 0.03	10.75 ± 2.50	0.21 ± 0.02	10.01 ± 5.05
C20:4n‐3c	1.10 ± 0.17	29.93 ± 4.56	0.59 ± 0.18	50.7 ± 15.2	0.22 ± 0.01	66.29 ± 3.92
C20:5n‐3c (EPA)	6.11 ± 1.13	166.8 ± 30.8	2.19 ± 0.43	186.7 ± 36.3	3.01 ± 0.10	903.8 ± 30.2
C21:5n‐3c	0.30 ± 0.08	8.24 ± 2.25	0.22 ± 0.05	18.68 ± 4.29	0.18 ± 0.02	55.20 ± 4.85
C22:5n‐3c	2.57 ± 0.15	70.18 ± 4.08	1.11 ± 0.24	94.7 ± 20.3	0.54 ± 0.02	160 ± 6.71
C22:6n‐3c (DHA)	12.94 ± 3.26	353.2 ± 88.9	3.94 ± 0.81	335.8 ± 68.7	3.39 ± 0.10	1,016.1 ± 30.5
**∑ PUFA**	**26.29 ± 5.30**	**717 ± 145**	**29.63 ± 6.66**	**2,524 ± 568**	**30.45 ± 0.97**	**9,134 ± 292**
**Total**		**2,729 ± 465**		**8,531 ± 2125**		**30,000 ± 922**
∑ n‐6	1.37 ± 0.27	37.36 ± 7.30	15.02 ± 3.65	1,281 ± 311	14.25 ± 0.42	4,275 ± 127
∑ n‐3	24.68 ± 4.95	673 ± 135	14.48 ± 2.98	1,234 ± 254	15.92 ± 0.53	4,777 ± 159
n‐6/n‐3	0.06		1.04		0.89	
AI	0.43		0.19		0.23	
TI	0.22		0.18		0.21	

Abbreviation: n.d., not detected.

^a^ Values are expressed as mean ± standard deviation.

^b^ The FA is not confirmed by a standard, only by NIST library search.

The monounsaturated n‐9 FA, OA, was the dominant peak found in farmed salmon and its feed and represents as much as 44% and 41% of the FA content, respectively. This corresponds well with previously published literature, which also report elevated contents of OA in farmed salmon in accordance with the increased amount of plant‐based ingredients in the feed (Friesen et al., 2015; Sprague et al., 2016). The intake of OA has been associated with potential beneficial effects in patients suffering from type II diabetes (Vassiliou et al., 2009). Furthermore, LA, and to a lesser extent C16:0, and ALA are present in large quantities in both farmed salmon and the feed. OA, LA, and ALA are most commonly found in plant sources, and together with C16:0 they are the main constituents in rapeseed oil (Sharafi et al., 2015). Rapeseed oil is one of the main ingredients in salmon feed in Norway today (Aas et al., 2019). Additionally, the feed contained greater proportions of EPA compared to farmed salmon (3.0% and 2.2% respectively) and lower proportions of DHA (3.4% and 3.9%, respectively).

As a direct consequence of the higher lipid content of the farmed salmon, it displayed higher concentrations of most FAs. However, similar concentrations of both EPA (167 and 188 mg/100 g fish muscle, respectively) and DHA (353 and 335 mg/100 g fish muscle, respectively) were found in wild and farmed salmon. Albeit similar concentrations, the proportions of these n‐3 FAs were three times higher in wild salmon (6% and 13% of the FA content, respectively) compared to the farmed salmon (2% and 4% of the FA content, respectively). The main peaks of wild salmon were C16:0, C18:1n‐9c, C20:1n‐9c, C22:1n‐9c, and DHA, which accounted for 64.5% of the total lipid content. These results correspond with a study by Olsen et al. (2013) who reported that these five FAs accounted for 65% of the FAs content in wild salmon.

Erucic acid (C22:1n‐9c), which has been associated with a health risk to children under the age of 10, was found at roughly twice the concentration in the wild salmon compared to the farmed salmon (241.5 and 124.8 mg/100 g fish muscle, respectively). The EFSA issued a report in 2016 recommending a dietary limit of 7 mg/kg body weight per day (Knutsen et al., 2016), which means that a child of 25 kg has a recommended limit of 175 mg erucic acid per day. By consuming 100 g of the fish subjected to testing, one would receive 242 mg and 125 mg from wild and farmed salmon, respectively. Thus, consuming wild Atlantic salmon would exceed than the recommended daily limit.

### 3.3 Comparison of SFA, MUFA, and PUFA in Atlantic salmon

The SFAs, MUFAs, and PUFAs are associated with different effects on human health. Contrary to SFAs, MUFAs and especially PUFAs are believed to have positive effects on human health, and recommendations for substituting SFAs with MUFAs and PUFAs are well established. An overwhelming number of studies have been conducted linking the substitution of SFAs with MUFAs and PUFAs to a decreased risk of CVD (Hooper et al., 2015; Kris‐Etherton & Krauss, 2020; Siri‐Tarino et al., 2015). However, even this is a debated topic, and newer research indicated no significant association between intake of SFAs and CVD (Krauss & Kris‐Etherton, 2020; Zhu et al., 2019).

The wild salmon was found to be the richest in SFAs. The SFAs constitute 26.3% of the total lipid content found in wild salmon, while only 15.0% for farmed salmon. However, due to the higher total lipid content of the farmed salmon, it displayed a much higher concentration of SFAs (1278 mg/100 g fish muscle), compared to wild salmon (718 mg/100 g fish muscle). The MUFAs compose the largest proportions in both wild and farmed salmon (47.4% and 55.4%, respectively). As expected, due to the higher lipid content of the farmed salmon, it displayed a much higher concentration of MUFAs (4725 mg/100 g fish muscle), compared to wild salmon (1293 mg/100 g fish muscle). Relatively similar proportions of PUFAs were observed in both wild and farmed salmon (26.3% and 29.6%, respectively). Furthermore, the FAs C16:0 and C18:0 constituted the majority of the total SFA content for both fish, while the FAs OA, C20:1n‐9c, and C22:1n‐9c were present in major quantities of the total MUFA content. The n‐3 FAs EPA and DHA constituted the majority of the total PUFA content in wild salmon; however, LA and ALA were the major constituents of the total PUFA content in the farmed salmon.

### 3.4 Comparison of n‐6 and n‐3 FA in Atlantic salmon

The n‐3 and n‐6 FAs exhibits different biological effects. The n‐6 FAs have a tendency of being proinflammatory, whereas the n‐3 FAs, like EPA and DHA, inhibit inflammation (Simopoulos, 2008). As a result of the higher lipid content, the farmed salmon comprised of higher concentrations of both n‐3 and n‐6 FAs compared to wild salmon. However, the wild and farmed salmon displayed similar proportions of n‐3 and n‐6 FAs (26.1% and 29.5%, respectively, of the lipid content). Whereas the wild salmon comprised of more n‐3 than n‐6 FAs (24.7% and 1.4%, respectively), the opposite was found in farmed salmon where slightly more n‐6 than n‐3 FAs (15.0% and 14.5%, respectively) was observed. The proportion of n‐6 FAs were ten times higher in farmed salmon compared to the wild salmon and are believed to be a result of the feed composition.

Judging by our results, consuming 200 g of fish fillets would provide 2470 mg of n‐3 and 2562 mg of n‐6 FAs from farmed salmon and 1346 mg n‐3 and 75 mg n‐6 from wild salmon. Due to their benefits to human health, the marine n‐3 FAs EPA and DHA are of particular interest. Eating a dinner portion (200 g) of fish fillets would provide 1040 mg and 1045 mg EPA and DHA from wild and farmed salmon, respectively. Thus, only 48 g of wild and farmed salmon would be necessary to satisfy the recommended daily intake of EPA and DHA set by the EFSA Panel on Dietetic Products, and eating salmon twice a week would satisfy the recommended weekly intake (EFSA Panel on Dietetic Products, 2012). Furthermore, the consumption of wild salmon would yield approximately equal amounts of EPA and DHA compared to the farmed salmon, however, at a lower energy intake due to the lower lipid content.

### 3.5 The fish lipid fractions

The lipids were fractioned into NLs, FFAs, and PLs and the identified FAs from each fraction is presented as percentages of the total area (area %) in Table 2. The proportions of SFAs, MUFAs, PUFAs, n‐3, and n‐6 FAs found in the different fractions of the fish are also provided. The NLs, comprising the triacylglycerides, were by far the most abundant in both wild and farmed salmon, composing a total of, respectively, 74.4 ± 5.4 and 86.9 ± 16.3% of the lipids. The PLs constituted the lowest proportions of the lipids in wild salmon (5.5 ± 1.2%), whereas the second lowest in farmed salmon (7.0 ± 0.6%). The FFAs, however, constituted a total of 20.1 ± 1.9 and 6.1 ± 0.4% of the lipids in wild and farmed salmon, respectively.

**TABLE 2:** Comparison of the FA composition of the lipid fractions (NL, FFA, and PL) in wild (*n* = 3) and farmed (*n* = 3) Atlantic salmon given as percentages of the total peak area

Fatty acid	Wild salmon composition (%)			Farmed salmon composition (%)		
	NL	FFA	PL	NL	FFA	PL
C12:0	0.071 ± 0.001^a^	0.09 ± 0.01	n.d.	n.d.	n.d.	n.d.
C13:0 (4,8,12‐trimethyl)^b^	0.09 ± 0.02	n.d.	n.d.	n.d.	n.d.	n.d.
C14:0	4.28 ± 0.15	3.70 ± 0.31	0.97 ± 0.12	2.05 ± 0.03	2.88 ± 0.06	0.62 ± 0.05
C14:0 (13‐methyl)	0.19 ± 0.01	0.15 ± 0.02	n.d.	0.05 ± 0.00	n.d.	n.d.
C14:0 (12‐methyl)	0.13 ± 0.01	0.10 ± 0.01	n.d.	0.03 ± 0.01	n.d.	n.d.
C15:0	0.42 ± 0.02	0.34 ± 0.02	0.23 ± 0.03	0.140 ± 0.003	0.26 ± 0.01	0.13 ± 0.01
C16:0	18.08 ± 1.00	22.96 ± 0.83	22.86 ± 1.82	9.49 ± 0.09	19.14 ± 0.53	20.62 ± 0.89
C17:0	0.57 ± 0.05	0.36 ± 0.05	0.53 ± 0.08	0.21 ± 0.01	0.32 ± 0.01	0.29 ± 0.01
C18:0	3.67 ± 0.18	5.43 ± 0.23	4.01 ± 0.40	2.59 ± 0.07	7.55 ± 0.23	1.57 ± 0.06
C20:0	0.10 ± 0.01	0.10 ± 0.01	n.d.	0.283 ± 0.004	0.220 ± 0.005	0.08 ± 0.01
C22:0	n.d.	n.d.	n.d.	0.08 ± 0.01	n.d.	n.d.
C24:0	n.d.	n.d.	n.d.	0.07 ± 0.01	n.d.	n.d.
**∑ SFA**	**27.62 ± 1.45**	**33.21 ± 1.49**	**28.60 ± 2.46**	**15.00 ± 0.22**	**30.37 ± 0.85**	**23.30 ± 1.02**
C16:1n‐9c	0.15 ± 0.02	0.13 ± 0.02	n.d.	0.137 ± 0.004	0.13 ± 0.01	0.12 ± 0.01
C16:1n‐7c	6.22 ± 0.23	4.43 ± 0.47	1.05 ± 0.16	2.32 ± 0.04	2.06 ± 0.03	0.46 ± 0.03
C16:1n‐5c	0.27 ± 0.01	0.30 ± 0.02	n.d.	n.d.	n.d.	n.d.
C17:1n‐7c	0.24 ± 0.01	0.22 ± 0.02	n.d.	0.098 ± 0.004	n.d.	n.d.
C18:1n‐12c	0.86 ± 0.29	0.92 ± 0.14	0.64 ± 0.16	0.17 ± 0.03	n.d.	n.d.
C18:1n‐9c	17.76 ± 1.27	11.52 ± 0.47	7.42 ± 1.04	44.65 ± 0.45	28.34 ± 0.42	11.04 ± 0.79
C18:1n‐7c	4.14 ± 0.97	3.44 ± 0.50	1.98 ± 0.40	3.04 ± 0.05	2.68 ± 0.03	1.78 ± 0.08
C18:1n‐5c	0.28 ± 0.03	0.27 ± 0.03	n.d.	n.d.	n.d.	n.d.
C20:1n‐11c	0.89 ± 0.10	0.48 ± 0.06	0.23 ± 0.03	0.19 ± 0.03	n.d.	n.d.
C20:1n‐9c	7.54 ± 0.72	3.85 ± 0.20	1.24 ± 0.20	3.24 ± 0.25	1.67 ± 0.10	0.31 ± 0.03
C20:1n‐7c^b^	0.26 ± 0.06	0.15 ± 0.03	n.d.	0.11 ± 0.01	n.d.	n.d.
C22:1n‐9c	8.07 ± 1.20	3.27 ± 0.20	0.30 ± 0.01	1.26 ± 0.29	0.46 ± 0.08	0.11 ± 0.01
C24:1n‐9c	0.60 ± 0.10	0.29 ± 0.07	n.d.	0.07 ± 0.01	0.26 ± 0.01	0.28 ± 0.01
**∑ MUFA**	**47.29 ± 4.99**	**29.27 ± 2.21**	**12.86 ± 1.99**	**55.27 ± 1.15**	**35.60 ± 0.69**	**14.09 ± 0.97**
C16:2n‐4c	0.27 ± 0.05	0.18 ± 0.02	n.d.	0.17 ± 0.01	0.20 ± 0.03	n.d.
C18:2n‐6c (LA)	1.08 ± 0.04	0.77 ± 0.03	0.34 ± 0.03	14.40 ± 0.12	13.88 ± 0.26	2.84 ± 0.16
C18:3n‐6c	n.d.	n.d.	n.d.	0.08 ± 0.01	n.d.	n.d.
C18:3n‐3c (ALA)	0.88 ± 0.06	0.67 ± 0.02	0.20 ± 0.02	6.23 ± 0.31	7.13 ± 0.41	2.45 ± 0.18
C18:4n‐3c	1.23 ± 0.18	0.79 ± 0.14	0.21 ± 0.01	0.49 ± 0.04	0.43 ± 0.03	0.13 ± 0.02
C20:2n‐6c	0.24 ± 0.03	0.15 ± 0.02	n.d.	0.774 ± 0.003	0.61 ± 0.01	0.30 ± 0.03
C20:3n‐6c	0.06 ± 0.01	0.05 ± 0.01	n.d.	0.18 ± 0.02	0.15 ± 0.02	0.20 ± 0.02
C20:3n‐3c	0.19 ± 0.02	0.14 ± 0.02	n.d.	0.34 ± 0.01	0.31 ± 0.01	0.18 ± 0.03
C20:4n‐6c	0.29 ± 0.04	0.55 ± 0.03	0.60 ± 0.08	0.13 ± 0.01	0.16 ± 0.00	0.56 ± 0.03
C20:4n‐3c	1.33 ± 0.15	1.05 ± 0.06	0.44 ± 0.03	0.56 ± 0.05	0.55 ± 0.04	0.49 ± 0.06
C20:5n‐3c (EPA)	6.32 ± 0.23	10.72 ± 0.66	8.15 ± 0.58	2.08 ± 0.06	4.30 ± 0.12	8.90 ± 0.24
C21:5n‐3c	0.38 ± 0.03	0.24 ± 0.02	0.31 ± 0.04	0.04 ± 0.02	0.116 ± 0.004	0.15 ± 0.02
C22:5n‐3c	2.67 ± 0.30	2.39 ± 0.31	2.84 ± 0.56	1.05 ± 0.04	0.66 ± 0.05	2.50 ± 0.25
C22:6n‐3c (DHA)	10.08 ± 0.95	19.81 ± 1.70	44.44 ± 1.78	2.61 ± 0.22	3.88 ± 0.24	39.46 ± 0.81
**∑ PUFA**	**25.00 ± 2.19**	**37.49 ± 3.05**	**57.53 ± 3.13**	**29.15 ± 0.92**	**32.38 ± 1.23**	**58.16 ± 1.85**
∑ n‐3	23.06 ± 2.02	35.79 ± 2.93	56.60 ± 3.02	13.40 ± 0.75	17.37 ± 0.91	54.26 ± 1.61
∑ n‐6	1.67 ± 0.11	1.52 ± 0.09	0.94 ± 0.11	15.57 ± 0.16	14.81 ± 0.29	3.90 ± 0.24

Abbreviation: n.d., not detected.

^a^Values are expressed as mean ± standard deviation.

^b^ The FA is not confirmed by a standard, only by NIST library search.

Our reported proportion of NLs in farmed salmon was comparable to a study by Tsoupras et al. (2018) that reported a proportion of NLs of 85%. Additionally, our results correspond with a study by Bell et al. (1998), which reported that wild and farmed salmon contained, respectively, 72% and 89% NLs. Halvorsen (2019) reported that the NL fractions constituted 83% and 97% of the lipids found in wild and farmed salmon, respectively, which are, respectively, 9 and 10 percentage points higher than the results of the present study. However, unlike the present study, the subcutaneous fat was sampled. The proportions of PLs in Atlantic salmon have been reported to vary highly in literature from 2%–40% (Halvorsen, 2019; Tsoupras et al., 2018, 2019). It has also been reported that FFAs constitute only 1% of the lipids found in farmed salmon, while 8% in wild salmon (Halvorsen, 2019; Ruiz‐López et al., 2015). However, the proportions of FFAs found were 2.5 and 6 times higher for, respectively, wild (20.1%) and farmed salmon (6.1%). The reason for this might be that the wild salmon was not frozen quick enough after capture to prevent the lipases in the muscles to initiate decomposition. Thus, some of the FAs from NLs might have cleaved from the glycerol backbone turning into FFAs by lipid hydrolysis (Shewfelt, 1981). It is also worth mentioning that the wild salmon might have been sampled at different periods of its life cycle, which would influence the results.

As shown in Table 2, the NL fractions closely resembled the FA profile and the proportions of SFAs, MUFAs, PUFAs, n‐3, and n‐6 FAs compared to the complete FA profiles found in their respective fish. This was due to the NLs displaying the largest proportions of the lipids. The FFA fractions were the richest in SFAs, whereas the PL fractions in PUFAs and the NL fractions in MUFAs. Analogous to the complete FA profile, the FAs C16:0 and C18:0 constituted the majority of the total proportion of SFAs within each respective fraction for both wild and farmed salmon, while the FAs OA, C20:1n‐9c, and C22:1n‐9c constituted the majority of the total proportions of MUFAs. The n‐3 FAs EPA and DHA were the major constituents of the proportions of PUFAs within each fraction of the wild salmon. This was also the case for the PL fraction of the farmed salmon. However, the PUFAs LA and ALA constituted the major proportions within the NL and FFA fraction. DHA alone constituted 44 and 39% of the total area of the PL fractions of wild and farmed salmon, respectively. The PL fraction was the richest in n‐3 FAs (57 and 54% of the total peak area in wild and farmed salmon, respectively).

Our results show higher proportions of n‐6 FAs in the NL and FFA fractions of the farmed salmon, where the n‐6 FA constituted 16% and 15% of the NL and FFA fraction, respectively, while only 4% in the PL fraction. This might be due to the lipid fraction of the feed primarily consisting of rapeseed oil, which is rich in n‐6 FAs, and has been reported to comprise 92% triacylglycerides (Zaderimowski & Sosulski, 1978). In contrast, the n‐6 FAs constituted approximately 2% of the NL and FFA fractions and 1% of the PL fraction in the wild salmon. These results correspond with the findings of Halvorsen (2019).

### 3.6 Nutritional quality indices of the lipids

The n‐6/n‐3 ratio of the modern Western diets have been estimated to be 15–17/1 (Simopoulos, 2008). A high imbalance in the n‐6/n‐3 ratio has been linked to many chronic diseases, including coronary heart disease and CVD (Simopoulos, 2008). However, the importance of this ratio is debated, and the FAO does not give any specific recommendations (FAO, 2010). For years, nutritionists have emphasized adding fish rich in n‐3 FAs to the Western diets, with the purpose of obtaining a more optimal n‐6/n‐3 ratio (Simopoulos, 2002). The calculated n‐6/n‐3 ratio of the farmed salmon (1.04/1) corresponds well with the findings of Aas et al. (2019) that reported values of approximately 1/1. However, this was considerably higher than that of wild salmon (0.06/1). The higher ratio of the farmed salmon reflects the increased use of vegetable oils in salmon feed. An n‐6/n‐3 ratio below 5/1 is considered beneficial for human health (Simopoulos, 2002; Yang et al., 2015). Thus, consumption of both farmed and wild salmon could contribute to reduce the n‐6/n‐3 ratio. Assuming that the Western diets are rich in n‐6 FAs, wild salmon therefore displayed a more beneficial n‐6/n‐3 ratio.

The calculated AI value for farmed salmon (0.19) was lower than that of wild salmon (0.43). However, relatively similar TI values were observed among the salmons with 0.18 and 0.22 for wild and farmed salmon, respectively. High AI and TI values (>1.0) have been reported to be detrimental to human health (Ouraji et al., 2009; Stancheva et al., 2014). The values in the present study were all lower than 1, which indicate that muscle tissue of both wild and farmed salmon is beneficial from a health perspective.

## 4 CONCLUSIONS

The results presented in this study highlighted the quantitative diversity of FAs for wild and farmed Atlantic salmon. Substantial differences between the lipid contents of wild and farmed salmon were observed (2.14% and 8.97% of fish muscle, respectively). As a result of the feeding regime, farmed salmon were richer in MUFAs (55.4%) and PUFAs (29.6%) than the wild counterpart (47.4% and 26.3% for MUFAs and PUFAs, respectively) and contained considerably higher amounts of the EFAs C18:2n‐6c (13.8%) and C18:3n‐3c (5.6%) as well as the MUFAs C18:1n‐9c (44.0%). Furthermore, farmed salmon were far richer in n‐6 FAs (15.0%). In contrast, wild salmon was richer in SFAs (26.3%) and n‐3 FAs (24.7%). Additionally, the content of the marine n‐3 FAs EPA and DHA was almost identical in the wild and farmed salmon (520 and 523 mg/100g fish muscle, respectively). The proportions of the three fractions were 74.4%, 20.1%, and 5.5% of total peak area in wild salmon, while 86.9%, 6.1%, and 7.0% in farmed salmon. The high contents of MUFAs and n‐3 PUFAs relative to SFAs, along with favorable n‐6/n‐3 ratios, and AI and TI values suggest that both the wild and farmed Atlantic salmon display nutritionally beneficial profiles. However, wild salmon displayed the most beneficial of the two. Furthermore, consuming wild Atlantic salmon would yield a lower total fat intake, thus suggesting a substitution from farmed to wild Atlantic salmon may prove nutritionally favorable.

## CONFLICT OF INTEREST

The authors have declared no conflict of interest.

## Data Availability

The data described in the manuscript can be found at Mendely data at: https://doi.org/10.17632/sph4m8hn2t.1.
